# “I am yet to encounter any survey that actually reflects my life”: a qualitative study of inclusivity in sexual health research

**DOI:** 10.1186/s12874-016-0193-4

**Published:** 2016-07-27

**Authors:** Elise R. Carrotte, Alyce M. Vella, Anna L. Bowring, Caitlin Douglass, Margaret E. Hellard, Megan S. C. Lim

**Affiliations:** 1 Centre for Population Health, Burnet Institute, 85 Commercial Road, Melbourne, VIC 3004 Australia; 2 School of Public Health and Preventive Medicine, Monash University, Alfred Hospital, Commercial Road, Melbourne, VIC 3004 Australia; 3 Melbourne School of Population and Global Health, The University of Melbourne, Level 4, 207 Bouverie Street, Melbourne, VIC 3010 Australia

## Abstract

**Background:**

Heteronormativity describes a set of norms and assumptions pertaining to heterosexual identities and binary gender. In 2015, we conducted our annual *Sex, Drugs and Rock’n’Roll* study, an online health survey of over 1000 Victorians aged 15–29 years. Feedback from participants suggested that our survey contained heteronormative language. In response to this, we aimed to make inclusive changes to our survey via consultation with young gender and sexually diverse (GSD) people.

**Methods:**

We conducted two semi-structured focus groups in Melbourne with a total of 16 participants (age range: 21–28 years). Participants were mostly cisgender women, and there were two transgender participants and one non-binary participant. Participants also had a range of sexual identities including lesbian, queer, bisexual, pansexual, and asexual. Focus group discussions were transcribed verbatim and analysed thematically.

**Results:**

Most participants indicated heteronormativity affects their lives in multiple ways, noting its impacts on access to sexual healthcare, invalidating sexual experiences and miscommunication in forms and surveys. Overall, participants emphasised the need for sexual health research to avoid assumptions about behaviour, to be clear and eliminate question ambiguity and avoiding treating gender as binary. Participants also discussed how the *Sex, Drugs and Rock’n’Roll* survey could address a range of sexual behaviours and experiences, rather than focusing on penetrative sex, which many participants found invalidating.

**Conclusions:**

Our findings have important implications for future health surveys aimed at general populations. We present recommendations that encourage research to be more inclusive to ensure data collection from GSD participants is respectful and rigorous.

**Electronic supplementary material:**

The online version of this article (doi:10.1186/s12874-016-0193-4) contains supplementary material, which is available to authorized users.

## Background

Gender and sexual diversity is an umbrella term relating to gender expressions not matching male and female gender norms (including transgender and non-binary identities), sexual identities, attractions and behaviours other than heterosexual, and intersex variations where reproductive or sexual anatomy do not fit typical male or female classifications. Although these identities are increasingly being recognised and celebrated, many gender and sexually diverse (GSD) individuals experience the effects of heteronormativity [1]. Heteronormativity describes a set of societal assumptions and norms which are based on heterosexual, cisgender^1^ experiences, influenced by social biases, privilege and stereotyping [1–3]. Heteronormativity can have negative impacts on the psychological well-being of GSD people, contributing to feelings of invisibility, invalidation and marginalisation [1].

Examples of heteronormativity can be found in multiple settings. School-based sexual education is largely heteronormative, often lacking representation and discussion of GSD people and their experiences [2, 4]. Qualitative research suggests that heteronormativity in sexual education legitimises homophobic bullying and contributes to the marginalisation of GSD people [4]. GSD people may also experience heteronormativity in healthcare settings, making it difficult to access appropriate care. Some physicians make assumptions about their patients’ gender, sexual identity and behaviour due to their lack of awareness or hesitancy to approach sensitive topics. However, GSD people have unique health needs, influenced by both sexual orientation and behaviour [5, 6]. For example, physicians may inaccurately assume their GSD patients are not at risk of sexually transmissible infections (STIs) if they do not report penetrative sexual intercourse [7–12], even though lesbians and bisexual women are more likely than heterosexual women to have ever been diagnosed with an STI [13]. Inadequate healthcare access can adversely impact the sexual health and mental wellbeing of patients and negative experiences may contribute towards low continuity of care and satisfaction with health providers [8, 14–16].

Considering unique sexual health needs and barriers to appropriate healthcare access, there is a need to conduct research into the sexual health of GSD people to inform policy and practice. While studies specifically targeting GSD individuals exist, (e.g. [17, 18]) general population studies should also include these communities in order to accurately reflect the prevalence and behaviours of GSD individuals and allow methodologically sound comparisons between groups [19]. However, it is common for researchers to make heteronormative assumptions about their participants’ gender identities and sexual orientation based on limited responses – for example, making assumptions about sexual identity based on behaviour or vice versa [20]. It is also common for researchers to conflate sex assigned at birth, gender identity and genitals, leading to the miscategorisation or exclusion of participants with intersex variations, transgender participants and participants with non-binary identities [20]. Further, sexual health research commonly relies on categorisation of sexual behaviours that focus on penetration and assume the presence of a penis [20]. These problems are also common in other forms of written communication, such as healthcare intake forms, where GSD patients often struggle to accurately communicate their gender and sexual information with limited response categories, causing frustration and miscommunication [21]. Sexual health surveys should be designed to minimise heteronormativity and allow all participants to accurately and respectfully answer questions.

Although there are no universally accepted best practice recommendations, several organisations and researchers have made recommendations for inclusive research methodology. Researchers Ansara and Hegarty [22] provide recommendations regarding gender diversity in the context of psychological research, recommending use of blank text boxes for recording participants’ gender and only asking for sex if relevant to research, and – if necessary to use categories – to include multiple, itemised gender options and the ability to select multiple options [22]. Meanwhile, the Gender Identity in U.S. Surveillance group (GenIUSS; a collaboration between researchers and GSD groups) and the San Francisco Department of Public Health provide recommendations for population-based surveys; both recommend a two-step approach (i.e., asking about sex assigned at birth and also gender identity). This approach has high sensitivity and specificity with adult populations; however, there is no clear evidence that this approach is appropriate for surveys including adolescents [23, 24]. OII (Organisation Intersex International) Australia recommends intersex status be asked as a separate question; people with intersex variations do not necessarily identify with the term ‘intersex’ in relation to gender and/or sex [25]. Regarding sexual orientation, the Sexual Minority Assessment Research Team (SMART) published recommendations for general research, and recommend asking about the three major dimensions of sexual orientation (identity, behaviour and attraction) separately if relevant to the research [26]; to contrast, the LGBTI Alliance recommends asking about attraction or behaviour rather than identity for young people who may still be forming identities [19]. However, no known inclusive language recommendations exist that are specific to youth sexual health research, and no known papers report details of consultations with young GSD people on these issues. Therefore, these recommendations were not directly applicable to our research.

In this study, we aimed to fill this gap in the context of the *Sex, Drugs and Rock’n’Roll* (SDRR) survey, an annual survey of Australians aged 15–29 years conducted by the Burnet Institute since 2005 [27]. From 2005 to 2014, this general and sexual health survey was conducted at a music festival, where participants self-completed a brief, paper-based questionnaire. The survey defined sex as ‘sexual intercourse’ or ‘penis in vagina or penis in anus’ before questions on sexual behaviour, a definition similar to that used in other sexual health research aimed at general populations. In 2015, the survey was conducted online. Researchers received several negative comments about the language of our survey, with comments about heteronormative language relating to sexual health and sexual risk behaviour questions, suggesting we failed to develop rapport with some participants. It is likely that participants felt more comfortable communicating these issues online rather than face-to-face. Further, many female participants in our survey reported sex in the past year with female partners despite the survey’s definition of sex (involving a penis). Many of these women ticked an option indicating no contraception was used the last time they had sex, resulting in data which incorrectly assumed their STI and pregnancy risks were comparable to unprotected, vaginal intercourse. Therefore, this study explored the perspectives of young GSD people on issues of heteronormativity, sexual health and experiences with research, with the aim of improving and making more relevant the language used in the SDRR survey and similar research.

## Methods

### Recruitment strategy

Recruitment notices were posted on Burnet Institute’s website and social media channels as well as the social media channels of a variety of organisations focusing on gender and sexual diversity. Recruitment notices were also emailed to relevant university student organisations. Inclusion criteria were being aged 18–29 years, with the invitation requesting ‘young women who identify as gay, lesbian or bisexual (or otherwise report sexual interest in women), transgender people, and/or other young members of GLBTIQ+ communities.’ Further information was provided and consent forms distributed upon expression of interest. Participation in the earlier SDRR survey was not required. Prior to the focus group, participants reported their gender, sexual identity and preferred pronouns to be used when reporting results and when personally addressed within the focus groups. Participants were reimbursed for their time.

### Focus group discussions

Two ninety-minute focus group discussions (FGDs) were held in Melbourne. FGDs have previously been effective for obtaining in-depth information with GSD people [1, 28, 29]. Topics for FGDs were decided a priori; a discussion guide was developed by the researchers. This guide was influenced by literature about heteronormativity and sexual health needs of GSD people, and the researchers’ experiences working with young people and minority groups on sensitive topics such as sexuality.

The FGDs were facilitated by one member of the research team (EC) and two other members of the team assisted each group (CD, ML and AV). As informed by the discussion guide, the FGDs began with open discussion about why the participants were interested in discussing inclusive language in sexual health research. The first half of the FGDs was dedicated to semi-structured discussion on key topics including gender, sex and sexuality, and assumptions others make about these topics and difficulties in written communication of these topics including surveys and healthcare forms. In the second half of the FGDs, participants were divided into groups of two to four and given printed copies of the SDRR survey’s questions related to gender and sexual behaviour (Table 1). Participants annotated the questions and provided both written and verbal suggestions for improving the language of the survey. Interviews were recorded and transcribed verbatim. Identifying information was removed prior to analysis, and all participants were assigned pseudonyms.

**Table 1:** Changes to the *Sex, Drugs and Rock’n’Roll* (SDRR) survey

Topic	2015 questions	Updated questions	Description of changes
Gender	What is your gender? Options: Male, female, transgender, other [option to specify]	What is your gender? (Please select all that apply) Options: Female, male, transgender, non-binary/genderqueer, other [option to specify]	Participants now have more flexibility in specifying their gender identity and can choose multiple options that describe them. Transgender participants can specify their gender identity rather than just noting they are transgender.
Intersex status	Not asked	Intersex is a term for people born with atypical physical sex characteristics. There are many different intersex traits or variations. Do you have an intersex variation? Options: Yes/No	Inclusion of intersex status.
Sexual identity	How do you identify yourself? Options: Heterosexual (straight), bisexual, gay/homosexual/lesbian, questioning, queer, other [option to specify]	How do you currently identify yourself? (Please select all that apply) Options: Heterosexual (straight), gay/homosexual/lesbian, bisexual, pansexual, asexual, queer, questioning, I don’t know/unsure, I don’t label myself, other [option to specify]	Question reworded to specify current sexual identity. Participants able to choose multiple terms. Additional options included.
Sexual behaviour	How old were you when you *first* experienced the following? (drop down box with age options) Deep kissing, touching a partner’s genitals with your hands, being touched on your genitals by a partner’s hand, giving oral sex, receiving oral sex, vaginal intercourse (penis in vagina), anal sex (penis in anus) *In the following questions, ‘sex’ means vaginal and/or anal sex.* How many people have you had sex with in your lifetime? In the last 12 months, how many males have you had sex with? In the last 12 months, how many females have you had sex with? (Categories for response ranging 0-51+)	*Questions are asked in relation to the following behaviours:* Touching a partner’s genitals with your hands, being touched on your genitals by a partner’s hand, giving oral sex, receiving oral sex, vaginal intercourse (penetration of vagina by penis), anal intercourse (penetration of anus by penis), *The following questions are asked for each behaviour:* How old were you when you experienced this for the first time? How many people have you done this with in the your lifetime? In the last 12 months, how many partners of each gender identity below have you done this with? (# male partners/# female partners/# other)	More options included for each sexual behaviour rather than just age of first experience. ‘Sex’ not defined before questions; rather, questions are worded more specifically. Question asked in table format. Participants only asked relevant questions (e.g., if they have never experienced vaginal intercourse, they will not be asked subsequent questions about vaginal intercourse).
STI risk behaviour	In the last 12 months how often did you use a condom with [regular/casual/new] sex partner/s? Categories for response: N/A no partner/s in past 12 months, always used a condom, usually (>50 %), sometimes (≤50 %), never used a condom with partner/s	In the last 12 months, how often did you use a condom during fellatio (mouth to penis, ‘blow jobs’)? In the last 12 months, how often did you use a glove, dam or other barrier during cunnilingus (mouth to vulva or vagina, ‘going down’)? *The following questions are related to penetrative sex (i.e., penetration of a vagina or anus with a penis).* In the last 12 months how often did you use a condom with [regular/casual/new] sex partner/s during penetrative sex? Categories for response: N/A No [behaviour/behaviour with relevant partner/s] in last 12 months, always used a condom/barrier, usually (>50 %), sometimes (≤50 %), never used a condom/barrier [with relevant partner/s]	‘Sex’ not defined before set of questions; rather, questions are worded more specifically. Questions asked for fellatio and cunnilingus as well as penetrative sex. If participants report not engaging in the specified sexual behaviour in the last 12 months in earlier questions, they will not be shown the related questions for the behaviour.
Contraception use at last penetrative sex	The last time you had sex, which form(s) of contraception did you or the person you had sex with use? (tick all that apply) Options: Condom, oral contraception (the pill), injection (Depo Provera), implant (implanon), emergency/morning after pill, withdrawal/pulling out, none, other [option to specify]	The last time you had vaginal intercourse (penis in vagina), which form(s) of contraception did you or your partner use? (Please select all that apply) Options: N/A (one of us was pregnant or trying to become pregnant), condom, oral contraception (the pill), injection (Depo Provera), implant (implanon), intrauterine device (IUD), diaphragm, hormonal ring, emergency/morning after pill, withdrawal/pulling out, other [option to specify], I don’t know, none of these	Only asked if reporting vaginal intercourse ever. More options added.

N.B. ‘I don’t wish to say’ is also an option for all questions in the both original and updated surveys

### Analysis strategy

Qualitative thematic analysis was performed on both transcribed data and written notes from participants. Coding was undertaken by one researcher (EC). After data immersion, open coding was undertaken to apply headings to concepts represented in the text. [30] Codes were consolidated into higher-level themes concentrating on the impacts of heteronormativity; themes were refined and organised through an iterative process based on repetition of topics, relationships between codes and relevance to the study aims. After the FGDs, the researchers discussed and compared the groups’ suggestions to help inform decisions relating to survey changes. Decisions for survey changes were also informed by existing recommendations from community based organisations and researchers (e.g. [22, 23, 26]).

## Results

### Participants

Sixteen participants (age range 21–28 years, mean age = 24.9 years) attended the FGDs. Seven participants attended the first FGD and nine attended the second. Participants’ listed gender identities included cisgender woman (*n* = 12), cisgender man (*n* = 1), transgender woman (*n* = 1), transgender man (*n* = 1) and non-binary (*n* = 1). Participants sexual identities included lesbian (*n* = 6), queer (*n* = 2), bisexual (*n* = 2), pansexual/bisexual (*n* = 1), pansexual/queer (*n* = 1), queer/gay (*n* = 1), gay (*n* = 1) and asexual (*n* = 1). One participant specified she identified ‘with’ (not ‘as’) queer.

### Thematic analysis

The first FGD focused on issues relating to transgender people, lesbians and bisexual women. The second FGD also focused on lesbians and bisexual women, but explored further about asexuality and the influence of popular culture on assumptions and attitudes on GSD people. Despite these minor differences, an overarching theme of both FGDs was heteronormativity and its significant impacts on various aspects of participants’ lives. The prevalence of heteronormativity (a major theme) and its impact on three areas of interest (sub-themes) are discussed briefly below, followed by a summary of suggestions for improving the SDRR survey.

#### Heteronormativity is everywhere

Participants identified examples of heteronormativity in multiple settings, such as in healthcare, sexual education, the workplace and even in brief interactions with strangers. It was described as more frequent than homophobia. Most participants noted assumptions by others (typically heterosexual, cisgender individuals) about their gender and sexual identities, their relationships, their bodies and their sexual experiences. Experiencing and challenging heteronormativity was described as exhausting, invalidating, frustrating and a ‘battle’. The impacts of heteronormativity in regards to three key concepts are described below.

##### Heteronormativity is a barrier to sexual healthcare

Participants described heteronormativity as a barrier towards sexual health, specifically sexual health-seeking behaviour. Most participants reported sexual health-related experiences and frustrations, including doctors ignoring or struggling to acknowledge sexual identities and practices. Even when health professionals were aware and accepting of participants’ gender and sexual identities, stigmatising language or assumptions about patients’ sexual experiences (such as assuming that bisexual and pansexual participants led hypersexualised lifestyles) were common and acted as a barrier to developing rapport and receiving appropriate sexual healthcare. Some participants, particularly lesbian and bisexual women, described dismissive or ignorant attitudes of doctors towards sexual experiences and STI risk behaviours if there is no risk of pregnancy. Transgender participants acknowledged the difficulties of understanding and communicating their sexual health needs to doctors who appeared uncomfortable or were unfamiliar with transgender experiences and bodies.

### Heteronormativity invalidates sexual experiences

Participants described a heteronormative societal assumption that ‘sex’ is penis-in-vagina or penis-in-anus intercourse, and anything other than this is not ‘sex’. This assumption was particularly distressing to participants who did not engage in penetrative intercourse regularly or at all.

I just think having to justify stuff… it’s just very frustrating… if it doesn’t tick these particular boxes, these one or two boxes, then that’s not ‘sex’. *Lisa, 22, lesbian, cisgender woman*

It can make you feel like your sex doesn’t really matter. *Abby, 23, pansexual/bisexual, cisgender woman*

My experience, my relationships are valid. They’re valid to me. *Laura, 23, lesbian, cisgender woman*

Participants described this assumption in many areas of everyday life. For example, female participants in relationships with other women described being inappropriately questioned about their sex lives by other people. Further, most participants reported frustration and anger at school-based sexual education, which they generally described as unhelpfully focusing on heterosexual vaginal intercourse and STI risk and failing to prepare them for their adult sex lives.

When asked to define ‘sex’, participants struggled to identify a specific definition. Participants indicated that sex is diverse, personal, usually intimate, and may not necessarily be reflective of traditional views, even in heterosexual encounters. Participants also discussed how experiences of sex and sexuality evolve with age and individual experiences, and focusing on penetrative sex fails to capture a spectrum of behaviours, thoughts, attitudes and experiences. While participants in both FGDs concluded that it was unhelpful to have a specific definition of sex or define it on behalf of others, they also acknowledged that they wanted their individual understandings of what constitutes sex to be respected.

### Heteronormativity facilitates text-based miscommunication

In most participants’ experiences, surveys and forms designed for general populations rarely represent lifestyles of GSD people. Written communications, particularly those that rely on selecting a single category, pose a challenge for participants who do not necessarily fit into provided categories.

I am yet to encounter any survey on any topic at all that actually reflects my life, and the activities that I do with my life. *Ivan, 28, gay, transgender man*

Participants reported specific challenges with completing forms with regards to gender and sex – including having no options that describe them, not knowing how to ‘best’ respond to questions, and trying to balance providing accurate information with information that actually describes their experiences. It was also noted that the common forced choice between ‘male’ or ‘female’ can be upsetting for transgender and non-binary people.

After being presented with the SDRR survey’s definition of sex, participants were asked whether they would answer questions regarding sexual behaviour in alignment with their own ideas and definitions or with the survey’s definition. Most participants agreed they would often, if not always, answer with their own understanding of sex in regards to sexual partners and risk behaviours. Participants noted that the inclusion of such a definition suggests that penetrative sex is the clinical and the only recognised definition of ‘sex’, indicating a lack of empathy. Some participants reported filling any available free-text answer boxes with comments about heteronormative language in order to inform researchers of their predicament.

I don’t go into a survey expecting that the options will make me feel comfortable, or will be easy… when they ask me about sexual behaviour, I will probably have to, like, sit there for fifteen minutes, thinking, “What answer should I put down?” *Taylor, 21, queer, transgender woman*

Participants unanimously reported strong negative feelings towards the survey’s definition of sex, which resulted in the experience of ‘othering’ and feeling excluded. These feelings can influence the participants’ relationship with the organisation that has developed the survey, making it difficult to develop rapport.

When [my idea of sex] isn’t reflected in a survey, or not legitimised, it also feels like that survey and whoever’s funding that survey – and a lot of the time that’s the government – doesn’t care about you, or your health, or your wellbeing. *Ivan, 28, gay, transgender man*

Regardless of negative experiences with surveys, several participants reported a willingness to participate in sexual health research, ideally if it were made more inclusive and empathetic. Participants emphasised that there is a lack of data about GSD communities, particularly transgender and non-binary communities, and they wish to give data accurately and ‘respectfully,’ and have research be translated into positive change.

I think it’s important just to raise it and say, “We’re here. We’re here and we’re queer.” [laughter] “Take notice and research us!” *Anna, 26, lesbian, cisgender woman*

### Summary of suggestions for improving our survey

Participants readily agreed that the language of the SDRR survey (Table 1) could be improved, but acknowledged that this was not a straightforward task, particularly as language evolves within GSD communities. Participants emphasised the need to identify the true aim of each question in order to help eliminate ambiguity and clarify the desired response.

“What are you actually asking of me?” I think is the thing. I mean, it’s really tough in research, because you don’t wanna manufacture answers, but… when I’m filling out a survey like this that I know is important… I wanna know what it actually is that you wanna know from me. Do you wanna know if I’m at blood transmission risk? Do you wanna know if, you know, I am engaging in a social culture? What part of my experiences with this do you wanna know about? *Leah, 23, identifies ‘with’ queer, cisgender woman.*

#### Gender/sex

Participants questioned whether researchers were interested in the gender of the participants (a cultural concept), or the participants’ sex assigned at birth (biological). It was noted that only being able to select one option for gender was limiting, particularly as ‘transgender’ is an adjective and not a gender identity. Participants suggested either replacing categories with a free text box so survey participants could describe their gender however they desired, or having more diverse options. It was suggested to also have ‘cisgender’ as an option (accompanied by a definition for those unfamiliar with the term), to recognise non-binary identities and intersex variations, and to specify male-to-female and female-to-male transgender if using categories. Another possibility included allowing selection of multiple options. Other questions in the survey were criticised for treating gender as a binary, particularly for reporting sexual partners. Wherever terms like ‘boyfriend’ and ‘girlfriend’ were mentioned, it was suggested we include ‘partner’ either additionally or as a replacement.

#### Sexual orientation

It was suggested that the question on sexual identity (Table 1) should use the word ‘currently’ as sexuality is fluid. Similar to the question on gender, participants suggested allowing selection of multiple options. It was noted as unrealistic to list every possible sexual identity but the most obvious omissions were asexual and pansexual; other suggested options included: ‘Never considered it’, ‘I don’t know’ and ‘I don’t label myself’. Other suggestions included randomising the order of the list (to eliminate a perceived hierarchy of sexual identities), modifying future questions based on the participants’ selected sexual identity, and updating the list each year with options listed in free-form text. Another suggestion was including questions about attraction in place of, or in addition to, questions about identity.

#### Sexual behaviour

The main criticisms of sexual behaviour questions were heteronormative language and dismissiveness of sexual behaviours other than penetrative sex. It was suggested that the definition of sex could be removed (with sexual behaviour of interest specified for each question), or modified to be more inclusive. Participants suggested lessening the focus on penetrative sex and including sexual behaviours such as masturbation, pegging,^2^ and specifying receptive vs. insertive anal sex.

Regarding condom questions, participants suggested we restructure to determine the reason why a participant may select never using a condom, e.g., no chance of pregnancy or low perceived STI transmission risk. Other suggestions were including a broader definition of sexual behaviour but listing more barrier methods of contraception, or including a penetrative definition but ensuring earlier questions are not dismissive of sexual behaviours.

### Changes to the survey

Our revised SDRR survey questions are summarised in Table 1; changes were based on the results of FGDs, discussions within the research team, other surveys on topics of gender, sex and sexual diversity (e.g. [17, 18]) and recommendations from various sources (e.g. [22, 23, 26]). These changes were made with consideration of the key challenges of survey-based sexual health research, including identifying accurate measures of behaviour, minimising participation bias (including social desirability) and comprehension problems, allowing participants a safe space to accurately report behaviours that may be of a sensitive nature, and treating sexuality with nuance and respect [20, 31]. Although inclusivity regarding intersex status was briefly discussed in the FGDs, we did not have any participants who identified an intersex variation, and our decision to include intersex status as a separate question was informed by recommendations from OII (Organisation Intersex International) Australia [25].

## Discussion

This study reports on results of FGDs with young GSD people regarding inclusive sexual health research methodology. Similar to research into healthcare needs of young GSD people [32], participants reported negative experiences with heteronormativity, and an overwhelming desire to be treated with respect, competence and to have their gender and sexual identities recognised in a variety of formats. FGD participants confirmed prior feedback that some of the language of the SDRR survey was heteronormative, invalidating and could result in inaccurate data. Participants were passionate and eager to have their concerns heard with regards to inclusive language, and expressions of interest outnumbered the allocated places in the FGDs. The main points raised in the FGDs included rethinking the survey structure to ensure a range of sexual risk behaviours are captured accurately, to avoid treating gender as binary, and to ensure questions asked in the survey are clear and inclusive. As described in Table 1, our updated survey includes recognition of more gender and sexual identities, a significant restructure of the sexual behaviour section (including addressing more sexual behaviours and related STI transmission risks), and re-wording of several questions.

Inclusion and wording of questions is dependent on a particular study’s research goals [33]. We believe our changes are most appropriate at this time for the SDRR survey’s scope, location and audience – that is, sexual health research aimed at young, general populations. Our recommendations relating to gender are similar to those suggested by Ansara and Hegarty [22] if using categorical response options. Our findings expand the existing literature by providing recommendations specific to inclusivity in sexual health research. Although previous research has discussed the impact of heteronormativity among GSD populations, (e.g. [1, 3]) this had not previously been discussed in the context of research. Our findings also provide a detailed rationale on how heteronormative questionnaires can invalidate and frustrate GSD participants in the context of their lives, which are already influenced by heteronormativity. These findings also indicate that inclusive, respectful language and survey structure can positively influence rapport.

Despite our changes, our survey is still unlikely to fully capture the complexities of identities and experiences of participants; we have maintained a largely category-based multiple-choice structure to ease data collection for our expected large samples (*n* > 1000). It was within the scope of the research to focus on gender rather than sex assigned at birth; for other studies, particularly those focusing on adult populations, the two-step process may be most appropriate [23, 24]. We have focused on identities, which may not capture experiences and attraction, likely leading to underreporting of some experiences (e.g., some people with transgender life experiences do not identify as ‘transgender’) [20, 23]. Of note, we have added a significant number of questions to our survey, increasing the risk of participant fatigue. Our survey is aimed at a general population; it is likely that some terminology used in the survey may be confusing for some participants. We aim to address this limitation by wording questions clearly [23]. We will review the content of the survey over time from a range of perspectives, and modify survey language if deemed necessary.

Revisions to the survey introduce some additional limitations regarding coding and analysis. Importantly, as our questions have been modified, we will no longer be able to directly compare data for some variables across earlier surveys. Although we have included more categories for gender and sexual identities, it is likely that prevalence estimates of some communities in our sample will be small. Sample size may limit data analysis strategies; for example, although we will ideally conduct analyses among a diverse range of gender identities rather than excluding ‘outliers’ [22], any analyses performed on these groups are likely to have relatively large margins of error and we may be unable to make meaningful conclusions about behaviour [31]. Initially, we may be required to collapse categories during data analyses (although prevalence can still be reported in text); this has occurred in prior research [17]. Depending on response rates, we will need to consider using appropriate coding schemes which do not code gender identities as mutually exclusive or treat sexual minorities as a homogenous group [20, 22]. It is possible that over time, we can combine data to increase the sample size of minority groups and perform meaningful analyses [23, 33]. Although not within the scope of our research, other sexual health surveys should consider over-sampling minority populations, such as specifically recruiting from GSD events or organisations, or use of strategies to maximise participation of minority groups, e.g., time-location sampling or respondent-driven sampling [33]. When deciding on the best approach to inclusivity, future studies will need to consider balancing issues such as budget, space limitations, data accuracy, target population demographics, and coding strategies.

## Conclusions

Based on our results, and with consideration of other methodology recommendations for general health research (e.g. [22, 23, 26]) and healthcare of GSD people in general [15, 34], we put forward recommendations for inclusive language in sexual health surveys involving general populations of young people (Table 2). Some recommendations, such as those related to gender and sexual orientation questions, are also relevant for more general research.

**Table 2:** Recommendations for inclusive sexual health research involving general populations

Topic	Recommendations
General	Eliminate question ambiguity by identifying the true aim of each question and the type of data you wish to collect. To achieve this, ensure questions are specific, clear and use defined time periods
	Avoid language that assumes participants are heterosexual or cisgender, or that certain behaviours are ‘normal’
	Use language that is culturally appropriate
	Avoid forced-choice, single option items and binary categorisations where possible
	Avoid skip patterns based on participants’ recorded gender or sexual identity; these require assumptions about participants’ attraction, identity, behaviour and genitals
	Routinely update questions based on cultural changes, feedback and relevant research while still considering data comparability across time
	Consult with GSD people and organisations if unsure of best wording of questions – avoid making assumptions
	Check relevancy and appropriateness of questions, particularly if space or your budget is limited, and aim to balance these considerations with making language as inclusive as possible
	Be aware of your own biases and knowledge limitations as a researcher
	Carefully consider all recommendations within the context of the research (including location and audience)
Gender/sex	Determine whether your research is interested in sex assigned at birth, gender identity, neither, or both
	If interested in sex *and* gender, consider a two-step process, i.e., asking for sex and gender separately
	When asking for participants’ gender identities, use open-ended or free text options if possible. If free-text is not an option, ensure options are more diverse than ‘female’ and ‘male’. Include recognition of transgender participants, non-binary/genderqueer participants, and other gender identities. Allow selection of multiple options or be specific with wording
	Ensure gender/sex questions are optional
	Avoid treating gender as binary (male/female) throughout the survey, including avoiding language implying a binary such as ‘both genders’ or ‘opposite sex’
	Avoid conflating gender and sex and be consistent with terminology
	Ask about intersex variations separately from sex and gender
Sexual orientation	Consider sexual orientation in terms of identity, attraction and behaviour, if relevant
	When asking about sexual identity, use open-ended or free text options where possible. If free-text is not an option, avoid ‘othering’ participants by including a range of sexual identities and options such as ‘I don’t know’ or ‘I don’t label myself’.
	Consider selection of multiple identities
Sexual behaviour	Avoid defining ‘sex’; instead, be specific to the sexual behaviour of interest
	Clearly identify when questions are referring to STI transmission risk, risk of pregnancy, or other objectives
	Consider appropriate skip patterns for questions that may be irrelevant to participants based on previous response patterns (this will also help reduce participant fatigue)
	Consider respectful and appropriate inclusion of sexual behaviours beyond penetrative sex

We believe that with careful consideration of the points raised in this project, investigators can make research significantly more inclusive, whilst maintaining rigour. It is unlikely that researchers will ever be able to be inclusive of every individual participating in their research; however, routinely recognising diversity in future sexual health research has several benefits. These changes have the potential to improve rapport with GSD participants. Further, these changes will allow for the collection of more accurate data which may inform evidence-based healthcare and health interventions.

## Abbreviations

FGD, focus group discussion; GLBTIQ+, gay, lesbian, bisexual, transgender, intersex, queer or other identities; GSD, gender and sexually diverse; STI, sexually transmissible infections

## Additional file

Additional file 1: 2015 Sex, Drugs and Rock'n'Roll survey. (DOCX 44 kb)

## Footnotes

1 Gender identity matches sex assigned at birth

2 A woman penetrating a man anally with a sex toy
