# Supplementary material for: “I am yet to encounter any survey that actually reflects my life”: a qualitative study of inclusivity in sexual health research
Source: BMC Med Res Methodol. 2016 Jul 27;16:86. doi: 10.1186/s12874-016-0193-4 (PMC4964098; doi:10.1186/s12874-016-0193-4)
Supplement: Additional file 1: — 2015 Sex, Drugs and Rock'n'Roll survey. (DOCX 44 kb) [file 12874_2016_193_MOESM1_ESM.docx]

**2015 *Sex, Drugs and Rock’n’Roll* survey**

**CONSENT FORM**

[Participant information and consent form]

I am aged 15-29 and consent to participate in this research.

- Yes
- No

**DEMOGRAPHICS**

1. What is your current postcode?
   - _ _ _ _
2. What is your month and year of birth?
3. How did you hear about this survey? (tick all that apply)

- An ad on Facebook
- A link a Facebook page
  - On which Facebook page or website did you hear about this survey? ____
- A link on a website
- A referral from a friend
- I don’t wish to say
- Other, ___________

1. Which of the following festivals have you attended in the last three years? (tick all that apply)
   - *List of mostly Victorian music festivals*
   - I don’t wish to say
   - Other, ___________
2. (if attended GTM) Did you see the I LOVE SAFE SEX message at Groovin’ The Moo? (as pictured). Tick all that apply

- Yes, I saw the message on the big screens on the Triple J and Channel V stages
- Yes I saw the message on on the big screens in the Moolin Rouge Tent
- Yes I saw the message on merchandise
- Yes I saw the message at the artist signing tent
- Yes I saw the message on stage being promoted by artists
- No I didn’t see the message
- I don't wish to say
- Other:

1. If you attended Groovin' The Moo, did you chat with the Red Aware team at the Condom Castle or around the event and learn anything about sexual health?

- Yes I did chat with them and I DID learn something new about sexual health
- Yes I did chat with them but I DIDN’T learn anything new about sexual health
- No, I didn’t chat with them
- I don't wish to say

1. What is your gender?

- Male
- Female
- Transgender
- Other, please specify ________
- I don’t wish to say

1. What country were you born in?

- Australia
- Other, specify ______
  - What year did you arrive in Australia? _ _ _ _
  - What is your current residential status in Australia?
    - Permanent resident/Australian citizen
    - Temporary resident (e.g. 457 visa)
    - Working holiday
    - International student
    - I don’t wish to say
- I don’t wish to say

1. What is the highest level of education you have completed?

- Did not complete high school
- Still at high school
- Completed high school
- Still studying: university
- TAFE, college or diploma
- Bachelor degree or higher
- I don’t wish to say

1. Who do you live with currently? (tick all that apply)

- Alone
- Parent(s)
- My partner
- Friend(s)/housemate(s)
- My child(ren)
- Other family
- I don’t wish to say

1. During a normal week, how much money do you have to spend on yourself for recreational purposes?

- Less than $40
- $40–$79
- $80–$119
- $120–$199
- $200–$299
- $300 or over
- I don’t wish to say

1. Are you an active member of any religious group or church?

- Yes
  - Please specify which religion or group you are affiliated with. ______
- No
- I don’t wish to say

1. Do you have your own Medicare card?

- Yes, I have my own card
- No, I just borrow the family card
- Not applicable
- I don’t wish to say

1. What is your *porn star* name?

First name = Name of first pet (if you’ve never had a pet, write your star sign)

Last name = Name of the first street you lived on

- Please write your answer here: _________________

**SEXUAL HEALTH**

**This section will ask you about your sexual health, knowledge of STIs and other related sexual health topics, and sexting.**

1. How do you identify yourself?

- Heterosexual (straight)
- Bisexual
- Gay/homosexual/ lesbian
- Queer
- Questioning
- Other, specify ____________
- I don’t wish to say

1. In the last 12 months have you visited a health service (e.g. general doctor’s clinic, sexual health clinic) for your health?
   - Yes
   - No
   - I don’t wish to say
2. Did you discuss sexual health and/or contraception with your doctor/practice nurse?
   - Yes
   - No
   - I don’t wish to say
3. When did you last have a test for STIs (excluding a pap smear)?

- I have never had one
- 0–3 months ago
- 4–6 months ago
- 7–12 months ago
- 1–2 years ago
- More than 2 years ago
- I don’t wish to say

1. These questions are to test your knowledge of Sexually Transmitted Infections (STIs), sexual health and other related topics:

|  | True | False | Don’t know | I don’t wish to say |
| --- | --- | --- | --- | --- |
| Chlamydia can be diagnosed by a urine test |  |  |  |  |
| Taking the contraceptive pill for a long time, without breaks, reduces your fertility in the future |  |  |  |  |
| Gonorrhoea, syphilis and chlamydia can all be easily treated with antibiotics |  |  |  |  |
| The pap smear can be used to diagnose all the main STIs |  |  |  |  |
| If left untreated chlamydia infection can last for years |  |  |  |  |
| The pill provides some protection against STIs |  |  |  |  |
| In Victoria, it is illegal to forward sexts on to other people |  |  |  |  |
| People infected with STIs almost always have some symptoms |  |  |  |  |
| Chlamydia can make women infertile (unable to become pregnant) |  |  |  |  |
| Using two condoms is safer than just using one |  |  |  |  |

1. How likely do you think you are to get any STI?

- Very unlikely
- Unlikely
- Likely
- Very likely
- I don’t wish to say

**SEXTING**

1. Have you ever done any of the following related to sexually explicit images (sexting)?
   - Sent a picture/sext of yourself to someone else (e.g. sending a sext to your partner)
   - Forwarded a picture/sext of someone who is not you to someone else (e.g. forwarding a picture of your boyfriend to your friend)
   - Received a picture/ sext sent directly from the person in the image (e.g. a picture from your partner)
   - Received a forwarded picture/sext from someone (e.g. your friend sends you a picture of his girlfriend)
   - I have not done any of these things
   - I don’t wish to say
2. The following questions are about your opinions on sexting.

|  | Strongly agree | Agree | Neither agree nor disagree | Disagree | Strongly disagree |
| --- | --- | --- | --- | --- | --- |
| Most people my age sext regularly |  |  |  |  |  |
| It should be illegal for people under 18 to sext |  |  |  |  |  |
| It should be illegal for people to pass on a sext without permission |  |  |  |  |  |
| It’s risky for a girl to send a naked picture of herself to someone |  |  |  |  |  |
| It’s risky for a guy to send a naked picture of himself to someone |  |  |  |  |  |
| If someone I’d just started seeing sent me a sext, I might show it to some friends |  |  |  |  |  |
| If my boyfriend/girlfriend sent me a sext, I might show it to some friends |  |  |  |  |  |

**SEXUAL BEHAVIOUR**

The following section will ask you about your sexual experiences and how old you were when you experienced each of them for the first time.

1. Deep kissing
2. Touching a partner’s genitals with your hands
3. Being touched on your genitals by a partner’s hand
4. Giving oral sex
5. Receiving oral sex
6. Vaginal intercourse (penis in vagina)
7. Anal intercourse (penis in anus)
   - [for each behaviour] I have never done this
   - I don’t wish to say
   - Ages 10-29

This section is a continuation of questions relating to sexual experiences and behaviours.

1. How many people have you had sex with in your lifetime?
   - None
   - One sex partner
   - 2–3 sex partners
   - 4–5 sex partners
   - 6–10 sex partners
   - 11–20 sex partners
   - 21–50 sex partners
   - 51+ sex partners
   - I don't wish to say

“Sex” means vaginal and/or anal sex.

1. Have you ever had sex that resulted in an unplanned pregnancy?

- Yes
  - At what age did you have your first unplanned pregnancy?
- No
- I don’t wish to say

1. In the last 12 months how many MALES have you had sex with?
   - No male partners in the past 12 months
   - One male partner
   - 2–3 male partners
   - 4–5 male partners
   - 6–10 male partners
   - 11–20 male partners
   - 21+ male partners
   - I don’t wish to say
2. In the last 12 months how many FEMALES have you had sex with?
   - No female partners in the past 12 months
   - One female partner
   - 2–3 female partners
   - 4–5 female partners
   - 6–10 female partners
   - 11–20 female partners
   - 21+ female partners
   - I don’t wish to say
3. In the last 12 months how often did you use a condom with REGULAR sex partner/s?
   - N/A: no regular sex partner/s in past 12 months
   - Always used a condom
   - Usually (>50%)
   - Sometimes (≤50%)
   - Never used a condom with regular partner/s
   - I don’t wish to say

REGULAR PARTNER means boyfriend/girlfriend, in a relationship

1. In the last 12 months how often did you use a condom with CASUAL sex partner/s?
   - N/A: no casual sex partner/s in past 12 months
   - Always used a condom
   - Usually (>50%)
   - Sometimes (≤50%)
   - Never used a condom with casual partner/s
   - I don’t wish to say

CASUAL PARTNER means any other partner e.g. f**k buddy, one night stand, friends with benefits, just seeing someone.

1. In the last 3 months how often did you use a condom with NEW sex partner/s?
   - N/A: no new sex partner/s in past 12 months
   - Always used a condom
   - Usually (>50%)
   - Sometimes (≤50%)
   - Never used a condom with new partner/s
   - I don’t wish to say

A NEW partner refers to a sex partner who you first had sex with in the last 3 months.

1. Please select all the forms of contraception that you or your partner at the time have used ever. Tick all that apply.
   - Condom
   - Oral contraception (the pill)
   - Injection (Depo Provera)
   - Implant (implanon)
   - Intrauterine device (IUD)
   - Emergency/morning after pill
   - Withdrawal/pulling out
   - None
   - I don't wish to say
   - Other, _______
2. Thinking about the last time you had sex, please select the form of contraception that you or your partner used. Tick all that apply.
   - Condom
   - Oral contraception (the pill)
   - Injection (Depo Provera)
   - Implant (implanon)
   - Intrauterine device (IUD)
   - Emergency/morning after pill
   - Withdrawal/pulling out
   - None, because we were trying to get pregnant
   - None
   - I don't wish to say
   - Other
3. Did you receive sex education at school?
   - If yes, please specify which year you were in when you first received sex education. If you cannot remember, please give us an estimate

- I never received sex education at school
- Yes, in grade one
- Yes, in grade two
- Yes, in grade three
- Yes, in grade four
- Yes, in grade five
- Yes, in grade six
- Yes, in year seven
- Yes, in year eight
- Yes, in year nine
- Yes, in year ten
- Yes, in year eleven
- Yes, in year twelve
- I don’t wish to say

1. Where have you sourced the most useful information related to sexual health? Tick all that apply.

- Parents
- Other family (e.g. siblings, cousins)
- Partner
- Friend
- School
- Doctor/clinic
- Websites
- Social media
- Apps
- Pornography
- Youth organisations
- None of these
- I don’t wish to say
- Other, specify ___

**Apps, gambling and pornography**

1. In the last 12 months which of the following apps have you used? (tick all that apply)

- Snapchat
- Tinder
- Grindr
- eHarmony
- Brenda
- RSVP
- OKCupid
- None of these
- I don’t wish to say
- Other, specify _______

1. Do you like/follow any of the following types of pages on Facebook, Instagram or Twitter? (Tick all that apply)

- Brand of cider (e.g. Rekorderlig, Kopparberg)
- Brand of beer (e.g. Carlton Dry, Corona)
- Brand of spirits (e.g. Smirnoff, Bundaberg)
- Brand of wine (e.g. Brown Brothers, Yalumba)
- Alcohol retailer (e.g. Dan Murphy's, Liquorland)
- Online betting (e.g. Sportsbet, Ladbrokes)
- Cleanses or Detoxes (e.g. SkinnyMeTea, juice detox)
- Diet plans or weight loss/fitness challenge (e.g. I Quit Sugar, Michelle Bridges 12WBT, Kayla Itsines Bikini Body Challenge)
- Weight loss/fitness motivation profiles (e.g. personal trainers, athletes, fitness models
- Other health related pages (e.g. Cancer Council) None
- I don't wish to say

1. What proportion of young males your age do you think consume/watch pornography?

- All
- Almost all (>80%)
- Most (>50%)
- Some (<50%)
- A few (<20%)
- None
- I don’t know
- I don’t wish to say

1. What proportion of young females your age do you think consume/watch pornography?

- All
- Almost all (>80%)
- Most (>50%)
- Some (<50%)
- A few (<20%)
- None
- I don’t know
- I don’t wish to say

1. How old were you when you first viewed pornography intentionally? This means you didn’t stumble across it accidentally.

- I have never viewed pornography
- <10
- Options 10-29
- I don’t wish to say

1. In the last 12 months, how often did you view pornographic material? (e.g. videos, images of a sexually explicit nature)?

- Never
- Less than monthly
- Monthly
- Weekly
- Daily/almost daily
- I don’t wish to say

1. How many times in the past seven days have you viewed pornography?

- ____

1. How many times in the past month have you viewed pornography?

- ___

1. How many times in the past year have you viewed pornography?

- ___

1. (if not *never)* How did you most commonly view pornography in the past 12 months?

- Streamed/downloaded on a mobile phone
- Streamed/downloaded on a computer
- A DVD
- Live webcam
- Magazines/books
- Other, specify _________
- I don’t wish to say

1. Who did you usually view pornography with in the past 12 months?

- On my own
- With a partner
- With a friend/friends
- Other, specify _______

1. Did you spend money on any of the following activities in the last 12 months? (tick all that apply)
   - Betting on sports
   - Betting on horses or dog races (but not sweeps)
   - Pokie machines or slot machines
   - Casino table games (e.g. blackjack, roulette)
   - Poker (e.g. at home, at a casino, online)
   - None of these
   - I don't wish to say
2. How often, in days, did you bet on sports in the past 12 months?
   - Please write your answer here: ____
     1. For example:
     2. One time this year = 1 day
     3. Once per month = 12 days
     4. Once per fortnight = 24 days
     5. Once per week = 48 days
     6. Twice per week = 96 days
     7. Three times a week = 144 days
     8. Every second day = 182 days
     9. Every day = 365 days
3. In the last 12 months, which of the following did you use to bet on sports? (tick all that apply)
   - Mobile phone apps
   - Online (on a computer)
   - At a casino
   - At a TAB/sports bet site
   - I don't wish to say
   - Other: ________
4. Thinking about the last 12 months, how much, in dollars, did you spend betting on sports each month, on average?
   - Please write your answer here:
5. Thinking about the last 12 months, how much, in dollars, did you spend betting on sports?
   - Please write your answer here:
6. How often, in days, did you bet on horse or dog races (not sweeps) in the past 12 months?
   - Please write your answer here:
     1. For example:
     2. One time this year = 1 day
     3. Once per month = 12 days
     4. Once per fortnight = 24 days
     5. Once per week = 48 days
     6. Twice per week = 96 days
     7. Three times a week = 144 days
     8. Every second day = 182 days
     9. Every day = 365 days
7. In the last 12 months, which of the following did you use to bet on horse or dog racing? (but not sweeps)
   - Mobile phone apps
   - Online (on a computer)
   - At a casino
   - At a TAB/sports bet site
   - At the races/track
   - I don't wish to say
8. Thinking about the last 12 months, how much, in dollars, did you spend betting on horses or dog races (not sweeps) each month, on average?
   - Please write your answer here:
9. Thinking about the last 12 months, how much, in dollars, did you spend betting on horses or dog races (not sweeps)?
   - Please write your answer here:
10. How often, in days, did you bet on the pokies in the past 12 months?
    - Please write your answer here:
      1. For example:
      2. One time this year = 1 day
      3. Once per month = 12 days
      4. Once per fortnight = 24 days
      5. Once per week = 48 days
      6. Twice per week = 96 days
      7. Three times a week = 144 days
      8. Every second day = 182 days
      9. Every day = 365 days
11. In the last 12 months, which of the following did you use to bet on pokies or slot machines?
    - Mobile phone apps
    - Online (on a computer)
    - At a casino or other pokie's site
    - I don't wish to say
    - Other:
12. Thinking about the last 12 months, how much, in dollars, did you spend betting on the pokies/slot machines?
    - Please write your answer here:
13. Thinking about the last 12 months, how much, in dollars, did you spend betting on the pokies/slot machines each month, on average?
    - Please write your answer here:
14. How often, in days, did you bet on casino table games (e.g. blackjack, roulette) in the last 12 months?
    - Please write your answer here:
      1. For example:
      2. One time this year = 1 day
      3. Once per month = 12 days
      4. Once per fortnight = 24 days
      5. Once per week = 48 days
      6. Twice per week = 96 days
      7. Three times a week = 144 days
      8. Every second day = 182 days
      9. Every day = 365 days
15. In the last 12 months, which of the following did you use to bet on casino table games (e.g. blackjack, roulette)?
    - Mobile phone apps
    - I don't wish to say
    - Online (on a computer)
    - At a casino
    - Other:
16. Thinking about the last 12 months, how much, in dollars, did you spend betting at casino table games (e.g. blackjack, roulette) each month, on average?
    - Please write your answer here:
17. Thinking about the last 12 months, how much, in dollars, did you spend betting at casino table games (e.g. blackjack, roulette)?
    - Please write your answer here:
18. How often, in days, did you bet on poker (e.g. at home, online, casino) in the last 12 months?
    - Please write your answer here:
      1. For example:
      2. One time this year = 1 day
      3. Once per month = 12 days
      4. Once per fortnight = 24 days
      5. Once per week = 48 days
      6. Twice per week = 96 days
      7. Three times a week = 144 days
      8. Every second day = 182 days
      9. Every day = 365 days
19. In the last 12 months, which of the following did you use to bet on poker?
    - Mobile phone apps
    - Online (on a computer)
    - At a casino
    - At someones house
    - I don't wish to say
    - Other:
20. Thinking about the last 12 months, how much, in dollars, did you spend betting on poker games each month, on average?
    - Please write your answer here:
21. Thinking about the last 12 months, how much, in dollars, did you spend betting on poker games?
    - Please write your answer here:
22. Thinking about the last 12 months: [On a scale with never, sometimes, most of the time, almost always and I don’t wish to say]
    - Have you bet more than you could really afford to lose?
    - Still thinking about the last 12 months, have you needed to gamble with larger amounts of money to get the same feeling of excitement?
    - When you gambled, did you go back another day to try to win back the money you lost?
    - Have you borrowed money or sold anything to get money to gamble?
    - Have you felt that you might have a problem with gambling?
    - Has gambling caused you any health problems, including stress or anxiety?
    - Have people criticized your betting or told you that you had a gambling problem, regardless of whether or not you thought it was true?
    - Has your gambling caused any financial problems for you or your household?
    - Have you felt guilty about the way you gamble or what happens when you gamble?

**ALCOHOL AND OTHER DRUGS**

The following section relates to alcohol and other drug use.

When we talk about a serve of alcohol, we are referring to a standard drink, which equates to 10ml of alcohol.

[Image illustrating standard drinks]

1. How old were you when you had your first full serve of alcohol? For example, a full serve refers to consuming a full drink on your own, as opposed to just having a taste of someone else’s drink.

- I have never drank alcohol
- <10
- Options 10-29
- I don’t wish to say

1. How often did your parents buy you alcohol when you were under 18?

- I never drank alcohol under the legal age
- Never
- Occasionally
- Often
- Always
- I don’t wish to say

1. In the last 12 months, how often did you have a drink containing alcohol?

- Never-[ skip following qs]
- Monthly or less
- 2 to 4 times a month
- 2 to 3 times a week
- 4 or more times a week
- I don’t wish to say

1. In the last 12 months how many drinks containing alcohol did you have on a typical day when you were drinking?

- 1 or 2
- 3 or 4
- 5 or 6
- 7, 8 or 9
- 10 or more
- I don’t wish to say

1. In the last 12 months how often did you have six or more drinks on one occasion?

- Never
- Less than monthly
- Monthly
- Weekly
- Daily or almost daily
- I don’t wish to say

1. a) In the last 12 months what is the highest number of drinks you have drunk in one session?

- 1 or 2
- 3 or 4
- 5 or 6
- 7–10
- 11–19
- 20 or more
- I don’t wish to say

b) In the last 12 months how often did you consume this amount?

- Less than monthly
- Monthly
- Weekly
- Daily or almost daily
- I don’t wish to say

1. In the last 12 months how often have you been unable to remember what happened the night before because of your drinking?

- Never
- Less than monthly
- Monthly
- Weekly
- Daily or almost daily
- I don’t wish to say

1. Have you or someone else ever been injured because of your drinking?

- No
- Yes, in the last 12 months
- Yes, not in the last 12 months
- I don’t wish to say

1. How often do you smoke cigarettes or other tobacco?
   - Never smoked
   - Ex-smoker (I don't smoke now, but I have smoked at least 100 cigarettes or equivalent in my lifetime)
   - Less than weekly
   - Weekly
   - Daily
   - I don't wish to say
2. Have you ever used illegal drugs?

- Yes
- No
- I don’t wish to say

1. Which of the following illegal drugs have you used in the last month? (tick all that apply)

- None in past month
- Acid/LSD/mushrooms
- Cocaine
- Ecstasy/MDMA
- GHB (Juice)
- Heroin
- Marijuana/cannabis/pot
- Ice/crystal methamphetamine
- Speed powder
- Other, specify _____________
- I don’t wish to say

1. [*For each drug used in the last month*] Thinking about the last six months, how often, in days, did you use [*each drug ticked*]? For example, once in the last six months = 1 day, one per month = 6 days, once per fortnight = 12 days, once a week = 24 days, twice a week = 48 days, three times a week = 72 days, every second day 90 days, every day = 180 days

- _____

1. [if used speed powder] How old were you when you first used speed powder?

- ____

1. How did you generally use speed powder in the last six months? (tick all that apply)

- Ingested orally
- Snorted
- Injected
- Smoked/inhaled
- Other, specify ___________

1. To the best of your knowledge, how much does speed powder cost at the moment per point?

- ______

1. [if used ice] How old were you when you first used ice/crystal methamphetamine?

- ____

1. How did you generally use ice/crystal methamphetamine in the last six months? (tick all that apply)

- Ingested orally
- Snorted
- Injected
- Smoked/inhaled
- Other, specify ___________

1. To the best of your knowledge, how much does ice/crystal methamphetamine cost at the moment per point?

- ______

1. In the last 12 months, have you used any of the following drugs/substances illicitly, not as directed or prescribed to someone else? (tick all that apply

- Diet pills
- Detox/laxative teas
- Steroids
- Antidepressants
- Benzos (Valium, Xanax)
- Viagra
- Codeine
- Other opiates (morphine, oxycodone)
- None of these
- I don’t wish to say
- Other, specify ____________

1. In the last 12 months, have you used any of the following illicit drugs/substances? (tick all that apply)

- None
- Mephedrone (M-CAT, meow meow) and other cathinone drugs
- 2C-B, 2C-I, 2C-E and other 2C drugs
- 25I-NBOMe and other NBOMe drugs (which have been sold as ‘legal’ LSD)
- DMT
- Synthetic cannabinoids (such as Kronic)
- Other, specify ___________
- I don’t wish to say

MENTAL HEALTH AND BULLYING

1. In the last six months have you been the victim of bullying? (tick all that apply)

- Never in the last six months
- Yes, homophobic/transphobic
- Yes, racial
- Yes, physical
- Yes, cyber (online, mobile phone)
- Yes, school or university
- Yes, workplace
- Yes, other _______
- I don’t wish to say

1. In the last six months have you had any mental health problems? This includes any issues that you haven’t spoken to a health professional about.

- Yes
- No
- I don’t wish to say

1. [if yes] Could you please specify what this mental health problem/s was? (tick all that apply)

- Anxiety disorder (e.g. Generalised Anxiety Disorder, Obsessive Compulsive Disorder)
- Mood disorder (e.g. Depression, Bipolar Disorder)
- Eating disorder (e.g. Anorexia Nervosa, Bulimia)
- I don’t wish to say
- Other, specify ___________

1. How would you rate your overall mental health?

- Excellent
- Very good
- Good
- Fair
- Poor
- I don’t wish to say

1. Have you used anti-depressants in the last month?

- Yes
- No
- I don’t wish to say

**Finished**

You have almost reached the end of this survey. Thank you for your time and responses!

1. Would you like to enter the draw to win a [*company*] gift card? (As a reminder, your email will not be stored with your survey responses).

- Yes,
  - Please enter your email address below for the opportunity to enter the prize draw
- No

1. Would you like to be sent a summary of the results from this survey? (Approximately six months after the survey has closed). As a reminder, your email will not be stored with your survey responses.

- Yes,
  - Please enter your email address below to receive a summary of the survey results
- No
